# Build a Robust Learning Feature Descriptor by Using a New Image Visualization Method for Indoor Scenario Recognition

**DOI:** 10.3390/s17071569

**Published:** 2017-07-04

**Authors:** Jichao Jiao, Xin Wang, Zhongliang Deng

**Affiliations:** School of Electronic Engineering, Beijing University of Posts and Telecommunications, Beijing 100876, China; wxsubic@bupt.edu.cn (X.W.); dengzhL@bupt.edu.cn (Z.D.)

**Keywords:** image feature extraction, indoor scenario recognition, HOG feature descriptor, feature visualization, sparse representation

## Abstract

In order to recognize indoor scenarios, we extract image features for detecting objects, however, computers can make some unexpected mistakes. After visualizing the histogram of oriented gradient (HOG) features, we find that the world through the eyes of a computer is indeed different from human eyes, which assists researchers to see the reasons that cause a computer to make errors. Additionally, according to the visualization, we notice that the HOG features can obtain rich texture information. However, a large amount of background interference is also introduced. In order to enhance the robustness of the HOG feature, we propose an improved method for suppressing the background interference. On the basis of the original HOG feature, we introduce a principal component analysis (PCA) to extract the principal components of the image colour information. Then, a new hybrid feature descriptor, which is named HOG–PCA (HOGP), is made by deeply fusing these two features. Finally, the HOGP is compared to the state-of-the-art HOG feature descriptor in four scenes under different illumination. In the simulation and experimental tests, the qualitative and quantitative assessments indicate that the visualizing images of the HOGP feature are close to the observation results obtained by human eyes, which is better than the original HOG feature for object detection. Furthermore, the runtime of our proposed algorithm is hardly increased in comparison to the classic HOG feature.

## 1. Introduction

Scene recognition is a highly valuable perceptual ability for indoor navigation, unmanned vehicles, UAV, or other kinds of mobile robots. However, current approaches for scene recognition present a significant drop in performance in the case of indoor scenes. Early work in computer vision attempted to achieve scene recognition by using supervised classifiers that directly operate over low-level image features, such as colour, texture, and shape. The main problem with these approaches has been their inability to choose reasonable image features from complex scenes.

Sande and his colleagues used colour descriptors for object and scene recognition, which was efficient. However, this method was invariant to light intensity [1]. Pandey and Lazebnik utilized deformable part-based models for scene recognition, which outperformed some other state-of-the-art supervised approaches. However, this method cannot classify similar objects in low intensity indoor scenes [2]. Silberman and Fergus used a structured light depth sensor for assisting indoor scene segmentation. However, the depth sensor will fail to detect the accurate depth when the sensor is blocked by a wall or a pillar [3]. Li and Sumanaphan used the scale-invariant feature transformer (SIFT) to recognize bathrooms. However, they also indicated that the accuracy of their approach was low, making it difficult to detect a bathroom in low-resolution images [4].

According to our research, feature extraction is an important step in order to improve scene recognition. The histogram of oriented gradient (HOG) is an excellent feature that describes the local image texture information, which is widely used in pedestrian detection. However, a large amount of background noise is introduced while obtaining the abundant texture information using HOG features, which induces the computer to make wrong judgments.

In order to analyse the reasons for this, people began to understand the vision world of the computer from the view of a computer, which is achieved by visualizing image features. It is an important method for understanding the minds of computers and for locating the problems in computer vision. During our research into finding a useful feature for scene recognition, we found that it was difficult to find the reasons that result in errors in object classification and recognition. At present, visualization is a popular method for analysing progress and discovering the shortcomings of feature extraction methods [5,6].

The goal of information visualization is generally defined as providing useful tools and techniques for gaining insight into and understanding of the computer vision system or, more generally, to amplify cognition [7]. These are high-level cognitive issues that are difficult to measure with quantitative user studies. Tory and Moller, in their summary of expert reviews, recommend the use of visualization evaluation for analysing computer vision systems [8]. Moreover, inspired by further studies [9,10], we introduce this method to evaluate and find the problem resulting in recognition error by using the descriptor we built. Despite this encouraging progress, there is still little insight into the internal operation and behaviour of these complex models, or how they achieve such good performance. From a scientific standpoint, this is deeply unsatisfactory. Without a clear understanding of how and why they work, the development of better feature descriptors is reduced to trial and error. Therefore, we introduce the visualization approach for evaluating our proposed feature descriptor. In this paper, we introduce a visualization technique that reveals the input stimuli that excite individual image features. It also allows us to observe the evolution of features during detection and recognition in indoor scenarios. The visualization technique we propose uses a dictionary library and a new HOG-based feature to project the feature activations back to the input pixel space.

The rest of this paper is organized as follows: Section 2 discusses relevant previous work on the HOG-based method and the visualization-based evaluation method; Section 3 presents the mathematical framework behind our model to achieve HOGP extraction and its visualization; Section 4 presents an evaluation of the proposed method and a comparison with state-of-the-art approaches; Finally, Section 5 presents the main conclusions of this work and future avenues of our research.

## 2. Related Work

In recent decades, image classification technology has been widely used in the scenes and tasks of computer vision, which includes object recognition, scene classification, object tracking, pedestrian detection, and so on. Several of the most widely used feature descriptors are local binary pattern (LBP) [11], HOG [12], and scale-invariant feature transformation (SIFT) [13]. LBP features are usually applied to face detection and recognition [14,15]. HOG features are often used for pedestrian detection and tracking [12,16]. SIFT features are more widely used in target recognition and location. Moreover, the research for improving those descriptors has been achieved. Through the combination of HOG and LBP, the pedestrian detection accuracy was improved [17,18]. Additionally, PCA was used to reduce the dimensions and computational complexity of the SIFT feature descriptor [19]. These features and their improved versions are able to express the local image texture features. However, those feature descriptors were underdeveloped in image classification and recognition against complex backgrounds. Therefore, more details are needed to achieve high accuracy image classification and recognition. SIFT can express most of the image region information, but its computational complexity is still a problem. In contrast, HOG is more effective than SIFT in large-scale target detection, because HOG indicates less region information than SIFT. However, HOG is hardly beneficial in small-scale target detection [20]. According to our previous work, we found that image colour is useful in describing image features.

In this paper, we introduce PCA to extract the principal colour information from image pixels. Then the PCA information is combined with the HOG descriptor. Consequently, the new feature descriptor, named HOGP, can simultaneously contain the regional texture and colour information, which makes it clearer for expressing small and dim objects than other HOG-based methods. Trung and his co-authors project the HOG features into the linear subspace by PCA in order to locate the objects in images [21]. However, the runtime of this method will decrease for high-resolution images. López and his colleagues proposed an approach for vehicle–pedestrian object classification based on the HOG–PCA feature space, instead of the frame coordinates [22]. Savakis and his co-authors created an efficient eye detector based on HOG–PCA features that reduces feature dimensionality compared to the dimensionality of the original HOG feature [23]. Kim and his colleagues introduced the pedestrian detection system by using the HOG–PCA feature, which improved the detection rate [24]. Agrawal and Singh developed a novel framework for a face recognition system that outperforms in an unconstrained environment, in which the HOG–PCA feature was used [25]. Dhamsania and Ratanpara implied that action recognition can be estimated by computing the PCA–HOG descriptor of the tracking region in frames. The experiments were performed for two games: hockey and soccer [26]. However, those methods indicated that the computational time should be considered carefully.

Moreover, the image visualization method is widely used to evaluate the performance of image feature descriptors. In a further study [27], the authors proposed an image feature visualizing method for assessing their improved SIFT features by finding similar blocks in the input database and then seamlessly splicing and smoothing them. Vondrick visualized HOG features and convolutional neural network (CNN) features through a dictionary-based sparse representation method [28]. Mahendran visualized CNN features, SIFT features, and the features combined with CNN and HOG [29]. In their study [30], based on a qualitative visualization approach, Chu and his colleagues argued that the residual networks challenged our understanding that CNNs learn layers of local features. Therefore, we introduced the visualization method to evaluate the performance of the different features that are used in our experiment.

## 3. Algorithm

**Figure 1:**
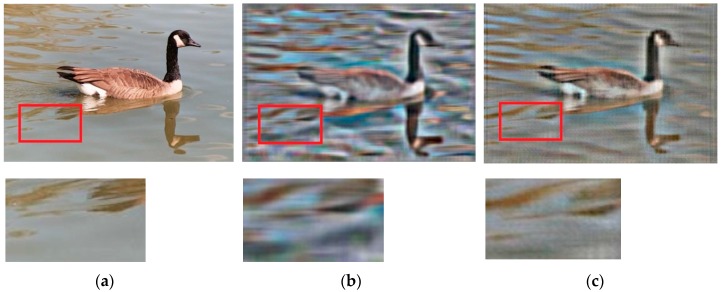
Comparison of our visualization with other visualization methods. (**a**) Original image; (**b**) HOG visualization [28]; and (**c**) HOGP feature visualization.

According to a previous study [28], we visualized Figure 1a. Then, we found that the contour detected by the computer was like a car from the computer’s perspective. Moreover, according to our test, the recognition result could not be changed, even by increasing the training samples or using other classification methods. After careful analysis, we suggested that the choice of the image feature descriptor did not distinguish the water waves and the car’s textures. That was because the Euclidean distance between the feature of the water wave and the feature of the car was closer than the distance between the feature of the water wave and other kinds of image features during the image classification. On the contrary, the human eye can easily distinguish the water wave from the noisy water surface.

**Figure 2:**
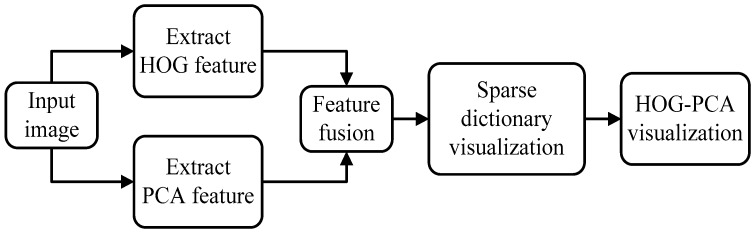
Flowchart of our proposed algorithm.

The flowchart of our algorithm is shown in Figure 2. In the first place, the HOG features are extracted from the input image. Meanwhile, the PCA features of the image’s colourful information are achieved [31]. After that, those two features are combined by cascade, which results in the formation of the HOGP features. Finally, the sparse dictionary method is introduced to visualize the HOGP features. It is noted that HOGP generation is inspired by Bajwa [32]. Bajwa argued that the pixel value of the image was a kind of feature and was also the most direct formation of the feature. Almost all of the image information can be expressed by the pixel values, which are named “direct features”, in our paper. The existing feature descriptors are representative and are powerful in certain backgrounds, but some of the image details are lost. In this paper, we propose HOGP for increasing feature robustness without increasing the computation cost in the feature extraction.

### 3.1. Grey Value

To increase illumination invariance and discriminative power, colour descriptors have been proposed recently [1]. Therefore, we obtain grey values of images by arranging the image’s block pixel values to a dimensional vector. We visualize direct features to validate that this feature could restore the image information to a high degree. The visualization method is briefly described as follows: 

Step 1: Build a dictionary library for the direct feature;

Step 2: Find the first p blocks with the smallest Euclidean distance by the K-nearest neighbour (KNN) algorithm that was proposed by a previous study [33];

Step 3: Compute the weighted average of the p image blocks to obtain a visualization image. 

According to our experimental results, the method can highly restore the colour information of the original image. However, it is clear that this colour feature does not have good robustness, but rather poor noise immunity. Moreover, the direct feature shows little reward in obtaining the gradient information, like the HOG-based feature. Therefore, we extracted the PCA information of the colour features, but did not use the colour features directly.

### 3.2. HOG Feature Descriptor

The HOG feature uses the image’s local gradient information to describe an image. The specific steps for extracting HOG features are outlined as follows:

Step 1: Normalize the gamma space to reduce the impact of the illumination change:

(1) $$I(x,y)=I{\left(x,y\right)}^{\lambda }$$

where *I* is the image, (x,y) is the location of a pixel, and λ is assumed to be 0.5 in this paper.

Step 2: Compute the image gradient:

(2) $$G(x,y)=\sqrt{{\left(I(x+1,y)-I(x-1,y)\right)}^{2}+{\left(I(x,y+1)-I(x,y-1)\right)}^{2}}$$

(3) $$\alpha (x,y)=\tan^{-1}\left(\frac{I(x,y+1)-I(x,y-1)}{I(x+1,y)-I(x-1,y)}\right)$$

where G(x,y) and α(x,y) represent the gradient magnitude and the direction of the pixel, respectively.

Step 3: Divide the image into cells whose size are $w_{cell}\times n_{cell}$. It is noted that each cell is composed of 8×8 pixels in our experiment. Additionally, the stride of the moving block was chosen to be eight pixels in our proposed method. The gradient directions of the region pixels are divided into eight bins, which are evenly spaced over $\left[0,180^{\circ }\right]$. According to the gradient directions, the gradient magnitudes of the regional pixels are voted into those eight bins. Then, the gradient magnitudes of the eight direction bins are added, and the bins are arranged to form an eight-dimensional feature vector.

Step 4: Combine 2×2 cells into a block. In this paper, each cell contains an eight-dimensional feature vector. Then, four cells that include eight-dimensional feature vectors are arranged to form a 32-dimensional feature vector.

In this way, the spatial relationship among pixels in a larger region is erected. The 32-dimensional feature vector is normalized to form a HOG feature.

### 3.3. HOGP Feature Descriptor

**Figure 3:**
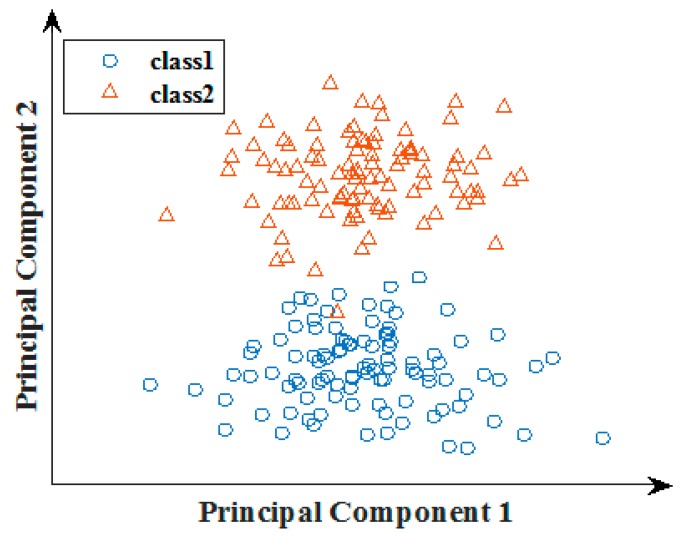
Projecting data in two orthogonal directions.

We extracted the PCA information of the pixel grey values. PCA provides better spatial information. PCA uses an orthogonal transformation to convert a set of observations of possibly-correlated variables into a set of values of linearly uncorrelated variables that are called principal components, which are shown in Figure 3. Therefore, in our work, we applied PCA along with the selection of maximum pixel intensity to create a new feature descriptor. The method yielded an image with less structural similarity to the source images along with low contrast and luminescence.

Let the dataset be an M×N matrix $D=\{X_{1},X_{2},\ldots ,X_{N}\}$, N is the number of samples, and each sample contains an M-dimensional feature vector $X_{i}=\{x_{1},x_{2},\ldots ,x_{M}\}$. The calculation of principal components consists the following three steps.

Step 1: Compute the covariance matrix C by using the following function:

(4) $$C=\frac{1}{N-1}(D-\bar{D}){\left(D-\bar{D}\right)}^{T}$$

Step 2: Obtain the transformation matrix W. First of all, the eigenvalues and eigenvectors of the covariance matrix C are computed. Then, eigenvectors are arranged according to the eigenvalues. In the following, the contribution rate of the eigenvalue is computed. Finally, the eigenvectors of the first K eigenvalues are selected to compose the PCA feature.

Step 3: Transform dataset D from an M-dimensional space to a K-dimensional space by transforming matrix W:

(5) $$Y=W^{T}D$$

where Y is a dataset after dimension reduction.

The $w_{pca}\times h_{pca}$ pixels corresponding to each HOG block are selected as the input dataset, $w_{pca}$ is the width of the HOG block, and $h_{pca}$ is the height of the HOG block. Each column of the dataset is a sample, and each pixel value is a feature. In other words, each sample includes $h_{pca}$ features. It is noted that both $w_{pca}$ and $h_{pca}$ are 16 in our experiment. 

Moreover, we find that the contribution rate of the first two-dimensional eigenvalues is commonly 80% larger after calculating the contribution rate of the eigenvalue of the dataset. Consequently, we select the eigenvectors’ first two-dimensional eigenvalues as the transformation matrix. After that, the dataset Y is called the PCA feature.

Finally, we combine the HOG feature with the PCA feature in cascade, in order to constitute a new feature named HOGP, by using Equation (6):

(6) $$HOGP_{p}(x,y)=\left\{Y_{p}(x,y),H_{p}(x,y)\right\}$$

where $Y_{p}(x,y)$ is the PCA feature of the pth image patch, and $H_{p}(x,y)$ is the HOG feature of the pth image patch. Therefore, we can find that HOGP is a coupling feature vector which includes two elements.

**Figure 4:**
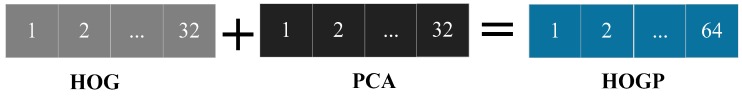
The framework for building a HOGP feature.

Equation (6) indicates that HOPG can achieve better performance than HOG in object recognition because of the introduction of the PCA feature. Therefore, compared to HOG, HOGP is more salient, which means that HOGP is more efficient than HOG in complex environments that include rotation, scale, and illumination. The process of creating a HOGP feature is shown in Figure 4.

### 3.4. Visualization Method

We use an effective sparse dictionary pair to visualize the image features. The specific steps are expressed as follows:

Step 1: Obtain the image features. Let I be an RGB original image which is divided into M patches $I=\{I_{1},I_{2},\ldots ,I_{M}\}$. m represents the pixel number of each patch $I_{i}$ that is an m-dimensional space. $P=f(I_{i})\in R^{n}$ means the image is packed into an n-dimensional descriptor vector. The transformation method is to extract image features. There are many methods to extract image features, such as HOG, SIFT, or HOGP.

Step 2: Construct a dictionary library. Our aim was to obtain the visualization image by using the image features, so we built a feature dictionary $FD=\{P_{1},P_{2},\ldots ,P_{K}\}$ where each element $P_{i}$ corresponds to an image patch, and K represents the number of the element. $ID=\{I_{1},I_{2},\ldots ,I_{K}\}$ is the image patch dictionary of the corresponding feature dictionary.

**Figure 5:**
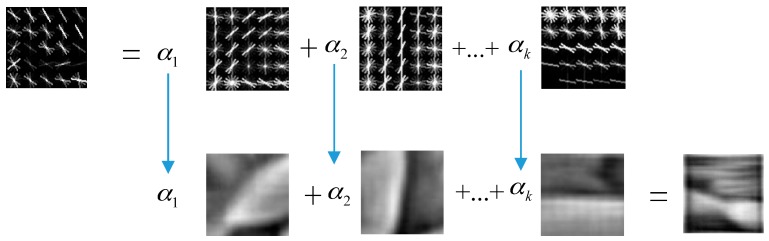
Process of the sparse representation of a dictionary pair.

Step 3: Complete the visualization by transferring the coefficients, which is shown in Equation (7). From Figure 5, we find that the combination of the features of each image patch that need to be visualized and the features in the dictionary are types of linear weighted processes.

(7) p=αFD,i=αID

where $\alpha =\{\alpha_{1},\alpha_{2},\ldots ,\alpha_{K}\}$ is a weight vector, p is the feature of a visualized image patch, and i is the visualization image.

We obtain the feature visualization by solving an optimization problem, which includes two optimizing procedures. One is to find out the coefficient through the feature dictionary:

(8) $$\begin{array}{ccc} \underset{\alpha \in R^{K}}{\mathrm{argmin}}{\left||\alpha FD-p|\right|}_{2}^{2} & & s.t.{\left||\alpha |\right|}_{1}\leq \varepsilon \end{array}$$

Another optimization is to use the obtained coefficient α to visualize the image patch. In order to express the process of the optimization problem, we re-wrote Equation (8) and the new function is shown as follows:

(9) $$\begin{array}{ccc} \underset{\alpha \in R^{K}}{\mathrm{arg min}}\sum_{i=1}^{M}\left({\left||\alpha FD-p|\right|}_{2}^{2}+{\left||\alpha ID-i|\right|}_{2}^{2}\right) & & s.t.{\left||\alpha |\right|}_{1}\leq \varepsilon \end{array}$$

According to Equation (9) we find that α can be obtained by minimizing the above function.

## 4. Results

### 4.1. Experiment Environment

**Figure 6:**
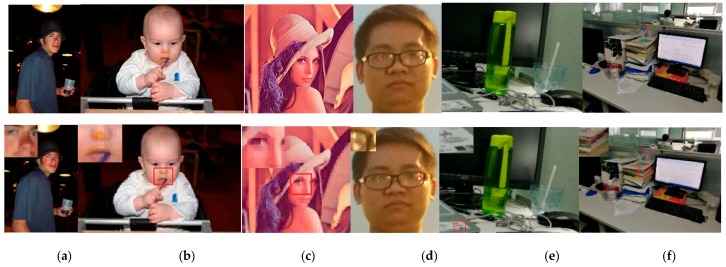
Direct feature visualization results. The upper row is the original image and the next row is the visualized image. (**a**) Man; (**b**) baby; (**c**) Lena; (**d**) boy; (**e**) desktop; (**f**) indoor.

We used a visualization method to assess the analysis of the experimental results. Part of our dataset comes from PASCAL [34] and another part comes from our collection based on a smartphone camera. Example images from the database are shown in Figure 6.

### 4.2. HOGP Feature Visualization

Based on the direct features, the visualization results of the direct image features are shown in Figure 6. According to Figure 6, we find that the method can highly restore the colour information of the original image.

From Figure 6, we find that the colour features coupled with PCA can recover a large amount of the original colour information. Figure 6a is the image that was used by a previous study [28], and this result is compared with the result obtained by HOGP. Figure 6b is a baby image selected from PASCAL. Figure 6c is the Lena image. Figure 6d is an image filled with noise due to being taken at night. Figure 6e is a desktop, where there is much foreground information and a simple background, since a simple background easily introduces noise into a visualization. Figure 6f is an indoor image. However, the shortcomings of this kind of feature are also obvious, which is that the regional connectivity is underdeveloped and the dimension is too high because of the lack of texture information.

### 4.3. Qualitative Results Based on Feature Visualization

Based on the sparse representation of the dictionary pair, we obtain the visualization results of the HOGP-based and HOG-based features. Comparing the HOGP feature visualization image to the HOG feature visualization image, the results indicate that HOG and HOGP features can both obtain rich texture information and have good robustness. However, HOGP features can highlight the theme and retain better colour information, which achieves better performance than the HOG features in image classification and recognition. Additionally, the HOGP features introduce less noise than the HOG feature. Therefore, the HOGP features can make the computer see objects more clearly than the HOG features.

### 4.4. Quantitative Evaluation in the Image Feature Matching Rate

As mentioned in the previous section of this paper, HOGP can improve performance in scene recognition. Therefore, in order to demonstrate the robustness of our proposed feature descriptor, HOGP, the image feature matching rate is introduced in our paper, which was inspired by a previous study [35]. Feature matching is the comparison of two sets of feature descriptors obtained from different images to provide point correspondences between images, which is shown in Equation (9).

(10) $$rate_{matching}=\frac{N_{corr}}{\min\left(n_{ref},n_{sen}\right)}\times 100\%$$

where $N_{\mathrm{corr}}$ is the number of the pair of matched features, $n_{ref}$ is the number of features of the reference image, and $n_{sen}$ is the number of features of the sensed image.

In this test, HOG and HOGP were used to extract the image features from the database. In our test, the existing matching method, named the Hamming distance, was implemented. Additionally, a statistically robust method, named RANSAC, was used to filter outliers in matched feature sets, which is useful in object detection.

**Figure 7:**
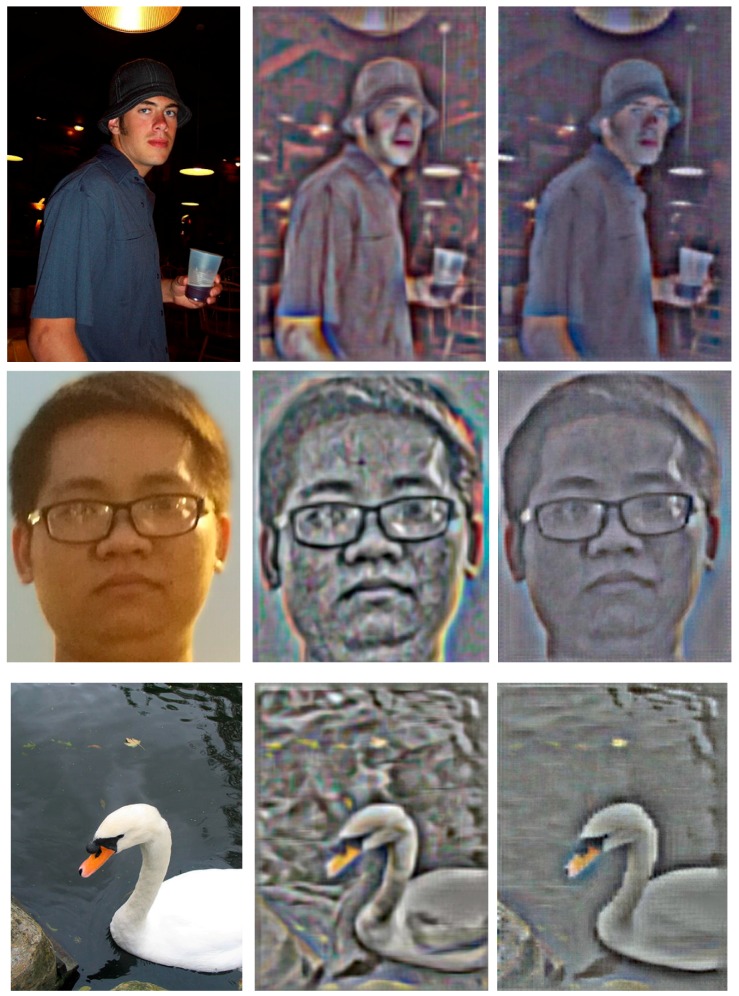

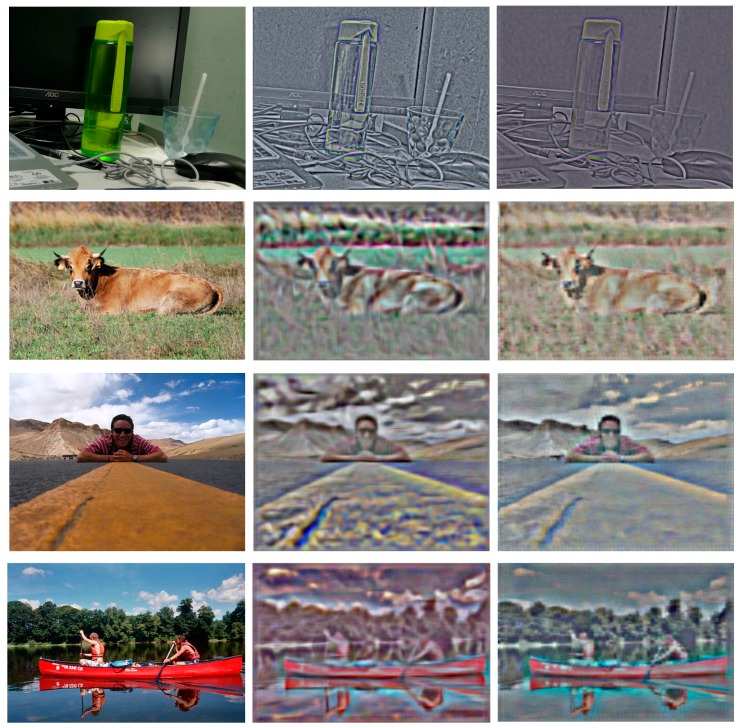
Comparison of the visualization results of HOG and HOGP features. The first column is the original image, the images in the second column are visualized by the HOG features, and the images in the third column are visualized by the HOGP features.

HOGP is robust to luminous intensity because it uses PCA to find key colour features. From Figure 7, we find that the middle figure was obtained by visualizing the HOG features of the left figure, and the right figure was obtained by visualizing the HOGP features. Comparing those two visualization results, we found that HOGP makes the computer see the image more clearly. From Table 1, we find that both HOG and HOGP obtained good performance in different scenes. Especially in pedestrian detection, the feature matching rate can achieve more than 76.3% with different illumination, which indicates that HOGP is robust to illumination change. Moreover, we find that HOGP achieved better performance than HOG in almost all of the scenes, even if the illumination intensity changed.

**Figure 8:**
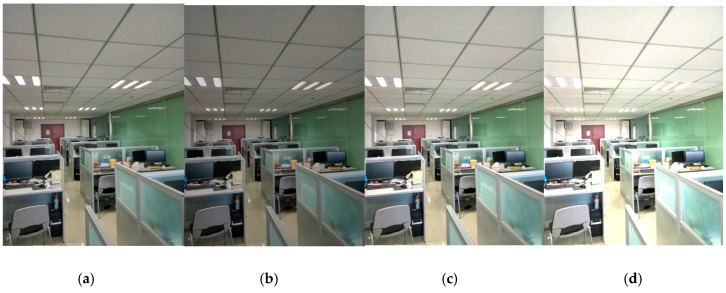
An example of an image with different luminous intensities. This is the indoor environment of our lab room. (**a**) Baseline; (**b**) 80% brightness; (**c**) 120% brightness; (**d**) 140% brightness.

It is noted that we divided our test images into four scenes: human facial, pedestrian, indoor environment, and animals. Figure 6 shows an example of our test image. In our test, each scene included 100 images. Moreover, different illumination changes were added to the test images. It is noted that three kinds of luminous intensity are added to our test image. Figure 8 displays an example of the images with different luminous intensity.

**Figure 9:**
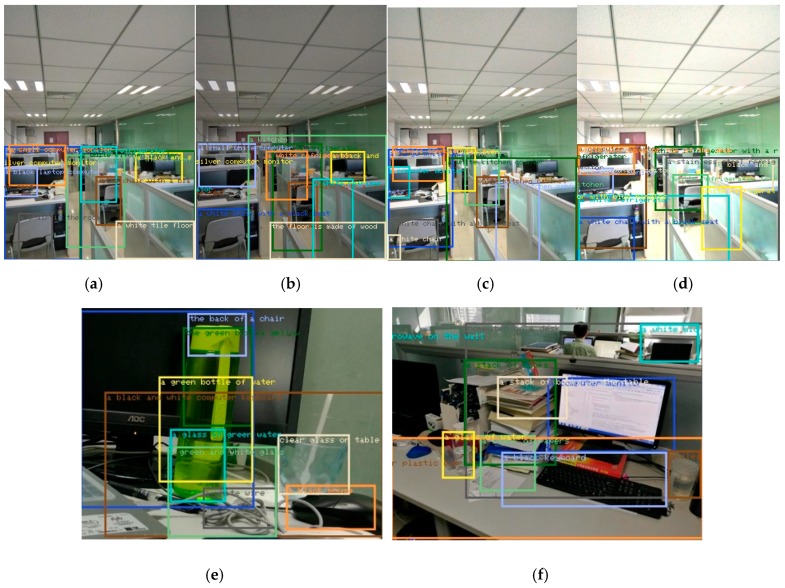
Object detection under different backgrounds based on our proposed method. (**a**) Baseline; (**b**) 80% brightness; (**c**) 120% brightness; (**d**) 140% brightness; (**e**) desktop object detection; (**f**) indoor object detection.

Given the feature-matching results, Figure 9 shows the comparison of the results between HOG and HOGP. Figure 9 shows the object detection results in different scenarios, which indicates that our proposed method can detect all of the objects in the images. Figure 9a–d implies that our HOGP feature descriptor is robust to illumination change. Figure 9e displays the performance of detecting similar and small objects based on HOGP features. Figure 9f gives the detection result in a complex indoor scenario that includes humans, computers, furniture, and so on.

**Figure 10:**
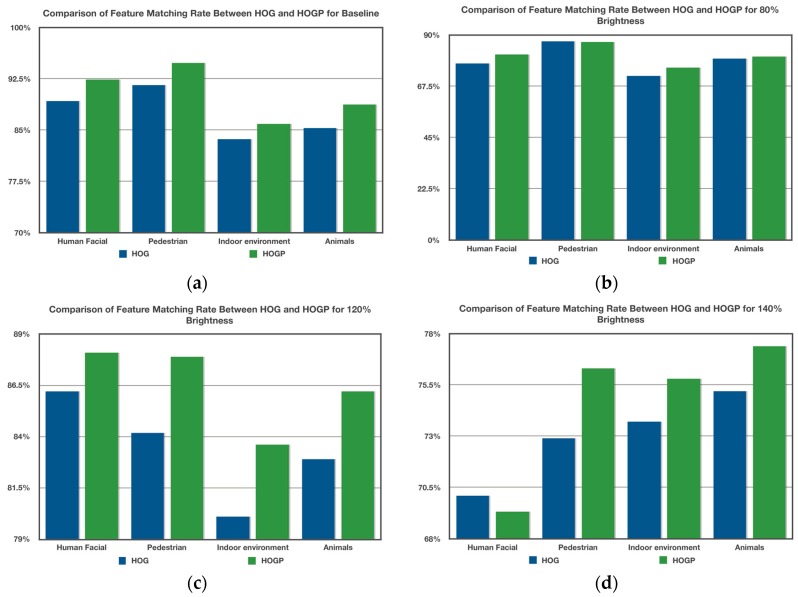
The comparison of results between HOG and HOGP for four kinds of scene. (**a**) Matching result for baseline; (**b**) matching result for 80% brightness; (**c**) matching result for 120% brightness; (**d**) matching result for 140% brightness.

A summary of the results is displayed in Figure 10 of four scenes with three kinds of luminous intensity. In the Figure 10 and Table 1, each point is the average of 100 independent runs with different random data samples. The horizontal axis represents the scene that was classified. The vertical axis represents the average results given by the feature matching score criterion. Figure 10a indicates that the larger the luminous intensity, the lower the feature matching rate. Those results imply that illumination change is an important factor that affects feature matching performance. Figure 10b shows that HOG obtained a better performance than HOGP only in pedestrian detection. However, the feature-matching rate of HOGP (86.9%) is close to that of HOG (87.3%). Figure 10d shows that HOG feature matching was 0.8% larger than that of HOGP in human facial detection when the brightness was 1.4 times that of the baseline, which implies that HOGP is more efficient than HOG.

**Figure 11:**
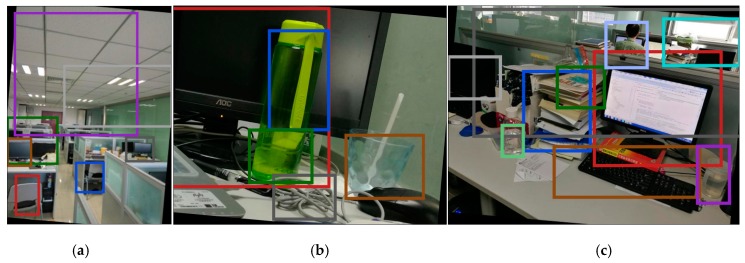
The object detection results using HOGP for rotated scenes. (**a**) Indoor scene; (**b**) desktop object detection; (**c**) indoor object detection.

**Figure 12:**
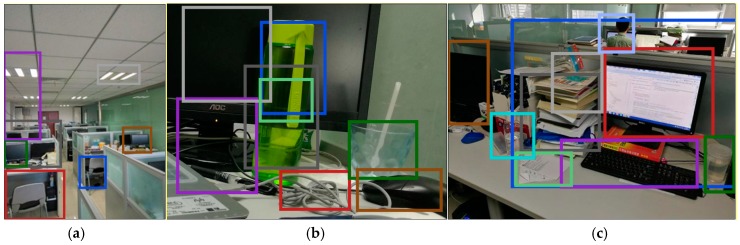
The object detection results using HOGP for scaled scenes. (**a**) Indoor scene; (**b**) desktop object detection; (**c**) indoor object detection.

Furthermore, we assessed our proposed method based on rotated and scaled images, which are used to improve the efficiency performance of HOGP in a complex environment. In order to improve the robust performance of HOGP, we rotated the test images with five different rotation values $\theta =\left\{5^{o},10^{o},15^{o},20^{o},25^{o},30^{o}\right\}$. Additionally, we scaled the test images with five different values κ={0.6, 0.8, 0.9, 1.2, 1.4, 1.5}. Figure 11 and Figure 12 show the object detection results for the rotated images ($\theta =5^{o}$), and scaled images (κ=0.8) by using HOGP, respectively.

**Figure 13:**
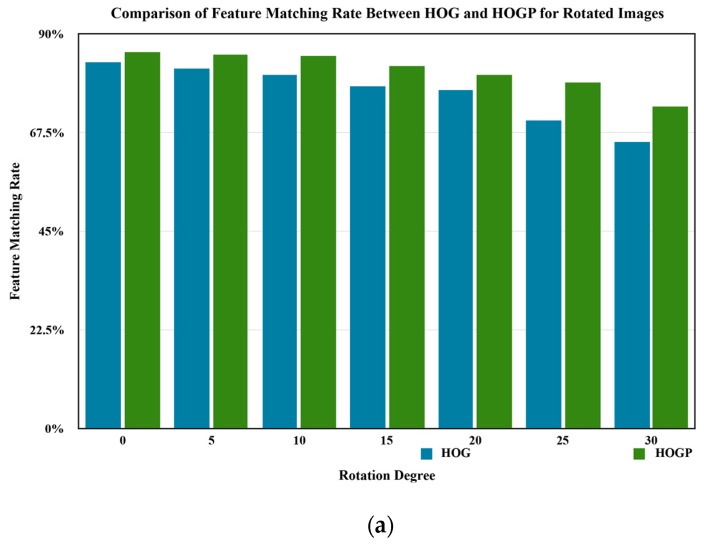

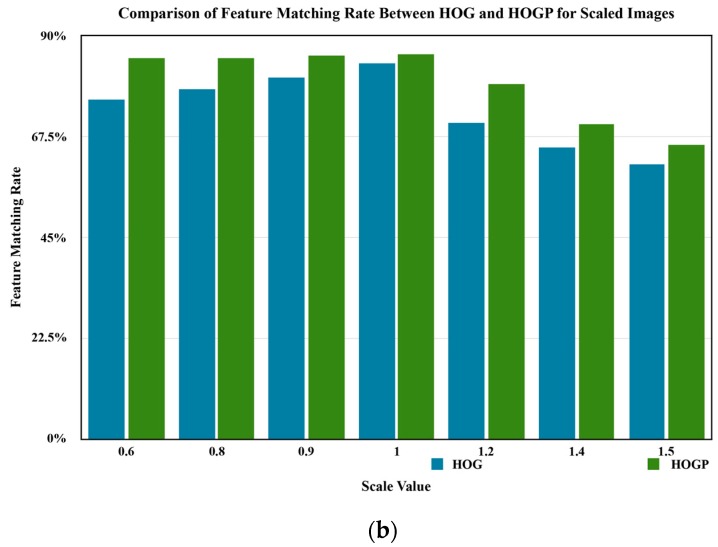
The comparison result between HOG and HOGP with respect to the feature matching rate. (**a**) Rotated images; (**b**) scaled images.

Figure 11 indicates the object detection results of the rotated images based on HOGP. Compared to Figure 9a,e, and f we find that all of the same objects in Figure 9 were also detected when the image was rotated to the right by $5^{o}$. Additionally, according to Figure 13a, we find that HOGP obtains better performance than HOG in all of the scaled images in the matching rate. Figure 12 displays the object detection results for the scaled images with κ=0.8. Compared to Figure 9a,e, and f, all of the same objects in Figure 9 were detected, even though the image is zoomed out. Furthermore, Figure 13b shows that HOGP achieves higher matching rates than HOG in all of the scaled images. Furthermore, Figure 13 also shows that the curve of HOGP’s matching rate is smoother than that of HOG, which implies that HOGP is more robust than HOG in object detection from scaled and rotated images. Considering these results, we can conclude that HOGP performs well and provides a robust and powerful alternative to object detection in complex scenes.

### 4.5. Runtime Comparison

In this section, we evaluate the running time of our algorithm by comparing it to state-of-the-art feature methods. Our implementation is coded in MATLAB. All experiments were done on a single core with 3.40 GHz Intel CPU with 12 GB of RAM.

In order to display the robust performance of our proposed method in object detection, we tested a number of different resolution images in different scenes, and the means of the runtime were calculated for HOGP and HOG. Table 1 shows the results for recovering the colour image from the original image. The runtime of the HOG feature compared to the HOGP feature increased by 4.07%. Consequently, we find that the runtime of HOGP has hardly increased, while the visualization result and the recognition rate is better than with HOG. Moreover, the runtime can be reduced to one-third for recovering the grey image. Comparing the runtime between the two descriptors, the HOGP-based algorithm is more efficient than the HOG-based method. This is because the runtime for extracting the direct image feature is reduced because PCA is used. Additionally, it is noted that the HOGP-based visualization results are better than that of the HOG-based method that was introduced in this paper.

## 5. Conclusions

To tackle the problem that HOG features introduce a greater amount of noise in complex scenarios, we proposed a new feature descriptor named HOGP. We used the visualization method to compare the performance of image features and intuitively understand the world that the computer sees. A qualitative and quantitative evaluation of the performance of HOG and HOGP under a variety of occasions was achieved. The experimental results showed that HOGP was more robust than other HOG-based features. Moreover, HOGP can make the computer see the image in the same way as the human vision system, which is proved by comparing the visualization performance to a state-of-the-art descriptor. In another word, HOGP can also improve the correct rate of computer classification and recognition. In the next work, we intend to apply HOGP to image classification and object detection after reducing the runtime of our proposed algorithm. 

## Figures and Tables

**Table 1:** Runtime comparison.

Pixel/Time(s)	Man (333 × 5000)	Desktop (1969 × 1513)	Lena (256 × 256)	Boy (443 × 543)
HOG + sparse dictionary	92.191	1423.114	36.532	123.521
HOGP + sparse dictionary	91.342	1378.683	29.563	111.049
